# Differentiating between subtypes of primary progressive aphasia and mild cognitive impairment on a modified version of the Frontal Behavioral Inventory

**DOI:** 10.1371/journal.pone.0183212

**Published:** 2017-08-16

**Authors:** Donna C. Tippett, Carol B. Thompson, Cornelia Demsky, Rajani Sebastian, Amy Wright, Argye E. Hillis

**Affiliations:** 1 Department of Neurology, Johns Hopkins University School of Medicine, Baltimore, Maryland, United States of America; 2 Department of Physical Medicine and Rehabilitation, Johns Hopkins University School of Medicine, Baltimore, Maryland, United States of America; 3 Department of Otolaryngology—Head and Neck Surgery, Johns Hopkins University School of Medicine, Baltimore, Maryland, United States of America; 4 Johns Hopkins Biostatistics Center, Johns Hopkins University, Baltimore, Maryland, United States of America; 5 Department of Cognitive Science, Krieger School of Arts and Sciences, Johns Hopkins University, Baltimore, Maryland, United States of America; University Of São Paulo, BRAZIL

## Abstract

Behavioral assessment has been investigated in frontotemporal lobar degeneration and Alzheimer’s disease, but has not been explored extensively in subtypes of primary progressive aphasia (PPA). We explored the ability of a modified version of the Frontal Behavioral Inventory (FBI-mod) to discriminate between patients with distinct subtypes of PPA and patients with mild cognitive impairment (MCI). We hypothesized that individuals with nonfluent agrammatic PPA (nfaPPA) would have higher negative behavior scores than other groups and that individuals with semantic variant PPA (svPPA) would have higher disinhibition scores than other groups. Family members and/or caregivers of 120 individuals with PPA and MCI (mean age 69.54+8.75 years; 65 (54%) female; education 16.06±2.68 years; disease duration 46.47±34.26 months) completed the FBI-mod [logopenic PPA (lvPPA) n = 40. nfaPPA n = 29, svPPA n = 27, MCI n = 24]. The groups were not significantly different in age, gender, education, or disease duration. There were no significant differences between the groups for negative behaviors (p = 0.72) and disinhibition scores (p = 0.14). When comparing negative and disinhibition scores (in percent), negative scores were significantly higher in all groups (p < 0.001). When comparing subtest items, there was a pairwise difference between lvPPA and svPPA for restlessness (lvPPA < svPPA, p = 0.02, after adjusting for multiple between-group comparisons). There was a significant difference in the proportion of severe neglect between the groups with lvPPA having a lower proportion than the other two variants (p = 0.05), and there was a significant difference in the proportion of severe poor judgment between the groups with lvPPA also having a lower proportion than nfaPPA (p = 0.04). This study reveals the greater negative behavioral disturbance than disinhibition in the PPA and MCI groups of similar age and duration since onset and identifies different profiles for some specific behaviors for the PPA groups. These findings may have clinical and practical implications.

## Introduction

Behavioral assessment has been investigated in frontotemporal lobar degeneration (FTLD) and Alzheimer’s disease (AD) (e.g., [1–4]) but has not been explored extensively in subtypes of primary progressive aphasia (PPA). In this study, the ability of a modified version of the Frontal Behavioral Inventory (FBI-mod) [5] was investigated to discriminate between patients with distinct subtypes of PPA and patients with mild cognitive impairment (MCI).

Primary progressive aphasia (PPA) is a language disorder characterized by insidious onset and gradual deterioration of language manifested by deficits in word finding, word usage, word comprehension, or sentence construction associated with atrophy of the frontal and temporal regions of the left hemisphere [6, 7]. In this collection of syndromes due to neurodegenerative disease, language is disproportionately impaired for at least two years, without impairment in other cognitive domains other than praxis [8]. PPA is comprised of three main variants, each with specific clinical features and pathophysiology: nonfluent agrammatic PPA (nfaPPA), semantic variant PPA (svPPA), and logopenic variant PPA (lvPPA) [9, 10]. Difficulty naming is an early and persistent impairment common to all three variants of PPA [11–13].

nfaPPA is characterized by core features of agrammatic language production and/or apraxia of speech [14–16]. Spoken modality-specific naming impairments are reported in nfaPPA [17] as well as naming deficits specific to impaired naming of actions rather than objects [17–19]. Individuals with nfaPPA may become mute early in their disease progression [20] and develop clinical features of parkinsonism and other symptoms of the underlying disease—usually corticobasal degeneration, progressive supranuclear palsy or frontotemporal lobar degeneration-tau (FTLD-t) [21]. Imaging abnormalities are typically present in left posterior frontal and insular regions [14, 22, 23]. In some cases, atrophy is present in the insula and premotor and supplementary motor areas [9, 10, 23]. Over time, there is progression of atrophy in nfaPPA into dorsolateral prefrontal cortex, inferiorly into superior temporal cortex, medially into orbital and anterior cingulate regions, and posteriorly along the Sylvian fissure into the parietal lobe [24]. In autopsy-confirmed cases of nfaPPA with tau-positive disease, there is inferior frontal and superior temporal cortical thinning [25]. Although nfaPPA is usually associated with tau-positive pathology, there is heterogeneity in the underlying pathology associated with this clinical syndrome. Non-tau pathology reported in nfaPPA include frontotemporal lobar degeneration-ubiquitin positive inclusions (FTLD-U) [26–27], Alzheimer’s disease (AD) pathology [28–30], frontotemporal lobar-TAR DNA binding protein (FTLD-TDP) [31–33], and TAR DNA binding protein 43(FTLD-TDP-43) [33].

svPPA is defined by marked anomia and single-word comprehension deficits across input and output modalities [34]. Individuals with svPPA may display progressively impaired object naming, with preserved naming of actions, and greater difficulty in the written versus spoken modality, although both modalities are compromised [18, 19]. This variant is associated with atrophy in ventrolateral anterior temporal lobes bilaterally, usually greater atrophy on the left [14, 23]. Speech fluency, syntax, and word repetition are preserved [14]. Individuals with svPPA also manifest behavioral symptoms as their disease progresses. Early symptoms include emotional distance, irritability, and disruption of physiologic drives; later symptoms are disinhibition and compulsions [35], which are symptoms of the most common underlying disease—FTLD-U [26, 28, 30] and its variant FTLD-TDP-43 [32]. Less commonly, svPPA is associated with AD pathology [29] and Pick bodies [36].

lvPPA is distinguished by word retrieval and phrase and sentence repetition deficits. Single word comprehension is relatively spared [10, 37]. Generalized cognitive decline, including language abilities, attention, memory, and visuospatial skills, is manifested over time [38]. Imaging abnormalities are seen in the left temporoparietal junction [14, 22]. Individuals with lvPPA often develop symptoms, such as impaired episodic memory, of the most common underlying disease—AD [9]. In addition to AD, Mesulam and colleagues [27] found that lvPPA is associated with FTLD-U.

Common behavioral manifestations of PPA include distress, sadness, and apathy, and secondarily changes in eating, aberrant motor behavior, agitation, disinhibition, and irritability [8, 39]. Behavioral manifestations of PPA have been compared in a few studies. On the Neuropsychiatric Inventory, individuals with semantic dementia demonstrated significantly more socioemotional behavioral dysfunction than the other variants of PPA and AD, specifically more disinhibition, aberrant motor behavior, and eating disorders-behaviors that are typical of FTLD and consistent atrophy of the anterior temporal lobes in svPPA. Behavioral profiles of the other PPA variants did not differ from each other or from AD in the type or severity of behavioral dysfunction. Behavioral abnormalities increased in severity with disease progression in semantic dementia, but not in the other PPA variants [40]. Similar findings were reported in a longitudinal study of behavior in PPA. On the Cambridge Behavioural Inventory Revised, individuals with svPPA exhibited significantly more behavioral disturbances of the type characteristic of behavioral variant frontotemporal dementia (bvFTD) compared with other PPA variants; individuals with nfaPPA showed loss of empathy [41]. In a comparison of svPPA and nfaPPA, those with svPPA were more agitated than those with nfaPPA, and those with nfaPPA were more depressed than those with svPPA. There were no differences in anxiety, irritability, apathy, perseverations, hyperorality, or abnormal motor behavior between these two variants [42]. Among individuals with PPA, those with svPPA have been found to have more severe damage to the uncinate fasciculus which is the major association pathway between the anterior part of the temporal lobe, including the amygdala, and the ventral frontal (orbitofrontal) region. Damage to this pathway has implications for a wide range of behavioral disturbances, such as apathy, impulsivity, and irresponsibility [43].

The Frontal Behavior Inventory (FBI) has been used extensively to distinguish individuals with bvFTD from those with AD [1–4]; vascular dementia [4, 44, 45]; depressive disorder [1]; and other FTD subtypes [1, 3, 4, 45, 46, 47, 48]. The FBI has been used in fewer studies to investigate behavioral manifestations in PPA. The FBI was developed to capture behavioral impairments in individuals with bvFTD [1, 3] and changes in behavior over time [46, 47]. The FBI is a caregiver questionnaire, consisting of deficit/negative behaviors (e.g., apathy, aspontaneity, indifference, inflexibility) and positive/disinhibition behaviors (e.g., perseverations/obsessions, irritability, excessive jocularity, poor judgment). Behaviors are rated on a 4-point Likert scale (0 = no change in behavior/no symptom; 3 = important change/severe symptoms). Each symptom is queried by asking informants questions requiring affirmative and negative responses if a symptom is present. Heidler-Gary et al. [5] modified the FBI such that only one question (the affirmative question) is asked about each symptom to achieve a more straightforward and less time-consuming interview.

Banks and Weintraub [49] compared patient and caregiver concepts regarding behavioral disturbances on a modified version of the FBI in 16 individuals with PPA, 10 individuals with bvFTLD, and 23 individuals with AD. There was better patient-caregiver agreement on the FBI in the PPA group compared to the bvFTD and AD groups, consistent with previous work [50]. In the PPA group, patient-caregiver ratings for apathy and aspontaneity differed significantly, reflecting patients’ loss of insight into these behaviors. The most frequently endorsed symptoms by individuals with PPA and their caregivers were logopenia, aphasia, inattention, apathy, disorganization, aspontaneity, and indifference. More recently, Konstantinopoulou, Aretouli, Ioannidis, Karacostas and Kosmidis [51] used the FBI to investigate behavioral disturbances in 30 individuals with AD and 87 with FTLD, including 19 with nfaPPA and svPPA. Individuals with PPA had higher (more pathological) ratings than those with AD on most FBI items, but lower scores than those with bvFTD. Those with PPA had the highest ratings on language related items, including logopenia, verbal apraxia, and comprehension compared to those with AD and bvFTLD. Ratings on non-language items were similar for those with PPA and bvFTD, including aspontaneity, inflexibility, disorganization, inattention, and hoarding. Heidler-Gary et al. [5] studied 30 individuals with AD and 50 with FTLD, including 25 with bvFTD, 13 with progressive nonfluent aphasia (an older term for nfaPPA), and 12 with semantic dementia (an older term for svPPA) to determine if the FBI-mod could assist in distinguishing AD and FTLD subtypes. There were significant between-group differences on the FBI-mod score overall and on the negative and disinhibition scores. Individuals with bvFTD had the most pathological FBI-mod scores. Scores were relatively normal on the FBI-mod for progressive nonfluent aphasia and semantic dementia compared to other groups, although there were deficits on other testing. Logopenia was reported in progressive nonfluent aphasia; apathy, indifference, inflexibility, perseverations, and hoarding were reported in semantic dementia.

The aim of this study was to investigate behavioral patterns in each of the clinical subtypes of PPA, which may have clinical and practical implications for families and caregivers. Our hypotheses were that individuals with nfaPPA would have higher negative behavior scores than other groups and that individuals with svPPA would have higher disinhibition scores than other groups.

## Materials and methods

### Participants

Prior to initiation of the study, the data collection, review and analysis were approved by the Johns Hopkins Medicine Institutional Review Board. Candidates for inclusion were individuals with PPA and mild cognitive impairment (MCI). One hundred twenty patients with PPA and MCI (mean ± standard deviation age = 69.54 ± 8.75 years; 65 (54%) female; mean education = 16.06 ± 2.68 years; disease duration 46.47±34.26 months) were enrolled. These individuals were evaluated in one author’s (AEH) outpatient cognitive neurology clinic and agreed to participate. Participants were diagnosed with PPA on the basis of presenting with a predominant and progressive deterioration in language abilities in the absence of major change in personality, behavior, or cognition other than praxis for at least two years [8]. PPA subtype was diagnosed by an experienced behavioral neurologist (AEH) based on medical history, comprehensive neurological examination, imaging, and a battery of language tests. Patients were classified using consensus criteria for each variant [10]. Patients were diagnosed with MCI, a cognitive state between normal aging and very early dementia in which there is objective memory impairment and other cognitive deficits; however, these deficits do not compromise daily function [52]. Most individuals with MCI presented with amnestic MCI or multidomain MCI. The MCI group was included as a comparison group to the PPA variants for negative behavior and disinhibition total scores.

### Materials

Family members and/or caregivers of these individuals with PPA and MCI completed the FBI-mod [5]. Informants were instructed to rate the extent of behavioral change for each symptom as “0” or “none”, “1” or “mild,” “2” or “moderate”, or “3” or “severe.” Caregivers were asked to base their responses on the extent of behavioral change for each item since the onset of symptoms. Negative behaviors included apathy, aspontaneity, indifference/emotional flatness, inflexibility, personal neglect, disorganization, inattention, loss of insight, and logopenia. Disinhibition included perseveration/obsessions, irritability, excessive jocularity, poor judgment, hoarding, inappropriateness, impulsivity, restlessness, aggression, hyperorality, hypersexuality, utilization behavior, and incontinence. A question accompanied each symptom to elucidate that behavior (e.g., Apathy: Has s/he lost interest in friends or daily activities?), and the total score from both types of behavior were calculated for each patient. Percent scores were calculated for each group by dividing the total scores by the total possible score for negative behavior (i.e., 27) and disinhibition (i.e., 39). Symptom scores were dichotomized into “3” (or “severe”) versus not severe ratings.

### Data analysis

Differences in the distribution of scores for negative behaviors, disinhibition, and total FBI-mod between PPA variants and MCI were evaluated by the Kruskal-Wallis rank test. Differences in the distribution of scores for each negative and disinhibition behavior between the PPA variants were also evaluated by the Kruskal-Wallis test. The Wilcoxon signed rank test was used to compare the difference in the negative behavior and disinhibition percent scores within diagnostic groups. Fisher’s Exact Tests were used to compare the proportion of “3” (“severe”) ratings between groups. Cronbach’s alpha was calculated for the negative and disinhibition behaviors for the PPA variant patients.

## Results and discussion

Table 1 describes the age, gender, education and disease duration of the groups. The groups were not significantly different on these characteristics.

**Table 1:** Age, gender, education, and disease duration for PPA subtypes, MCI, and for participants overall.

Variant	Age (yrs) (mean, SD)	Gender (F) N (%)	Education (yrs) (mean, SD)	Disease Duration (mos) (mean, SD)
lvPPA (n = 40)	70.38 (6.62)	24 (60)	16.78 (2.40)	50.85 (32.94)
nfaPPA (n = 29)	67.72 (7.03)	18 (62)	15.56 (2.55)	48.36 (35.35)
svPPA (n = 27)	67.93 (10.29)	15 (56)	15.56 (2.95)	40.38 (35.94)
MCI (n = 24)	72.17 (11.26)	11 (46)	15.60 (3.58)	42.79 (34.17)
Overall (n = 120)	69.54 (8.75)	65 (54)	16.06 (2.68)	46.47 (34.26)
P values*	0.196	0.395	0.226	0.623

F, female; SD, standard deviation; yrs, years; mos, months; lvPPA, logopenic primary progressive aphasia; nfaPPA, nonfluent agrammatic primary progressive aphasia; svPPA semantic variant primary progressive aphasia; MCI, mild cognitive impairment

*p values were calculated using one-way ANOVA for age, education, and disease duration and using chi square for gender.

### Comparison of negative behaviors, disinhibition, and total scores on the modified FBI between groups

**Fig 1:**
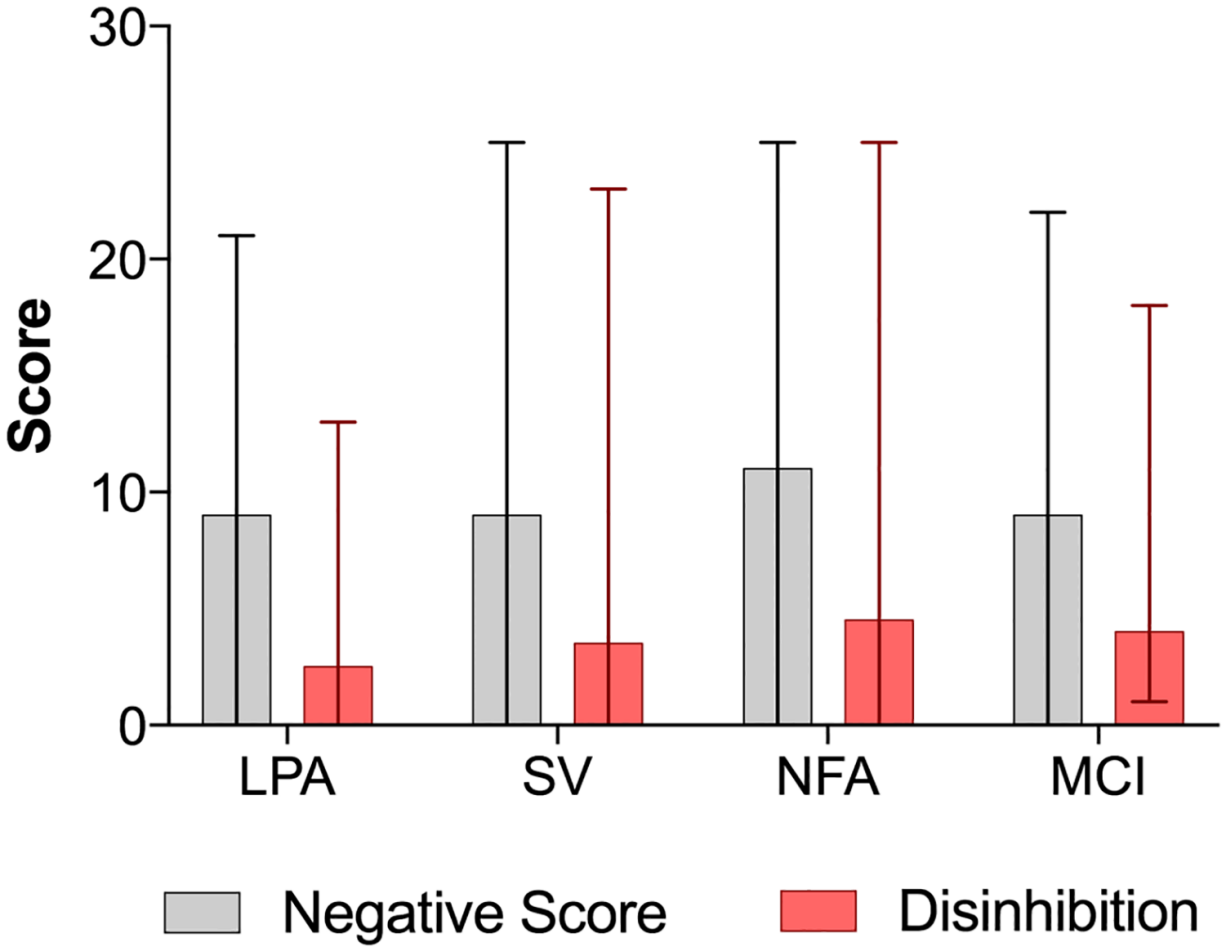
Medians and ranges for modified FBI negative behavior and disinhibition scores for PPA subtypes and MCI. lvPPA, logopenic primary progressive aphasia; nfaPPA, nonfluent agrammatic primary progressive aphasia; svPPA semantic variant primary progressive aphasia; MCI, mild cognitive impairment.

There were no significant differences in the distribution of negative behavior scores between the four groups (p = 0.72). The distribution of disinhibition scores was also not significantly different between groups (p = 0.14). Total FBI-mod scores were not significantly different between groups (p = 0.80) (Table 2; Fig 1).

**Table 2:** Comparison of negative behavior, disinhibition, and total scores on the modified FBI between PPA subtypes and MCI.

Variant	FBI-mod Negative Behavior Score (median, range)	FBI-mod Disinhibition Score (median, range)	Total FBI-mod Score (median, range)
lvPPA (n = 40)	9 (0–21)	2.5 (0–14)	13 (0–31)
nfaPPA (n = 29)	10 (0–25)	5 (0–25)	17 (0–46)
svPPA (n = 27)	9 (0–25)	4 (0–23)	13 (1–45)
MCI (n = 24)	9 (0–22)	5 (0–18)	13 (1–38)
Overall (n = 120)	9(0–25)	4 (0–25)	13 (0–46)
P values*	0.72	0.14	0.80

lvPPA, logopenic primary progressive aphasia; nfaPPA, nonfluent agrammatic primary progressive aphasia; svPPA semantic variant primary progressive aphasia; MCI, mild cognitive impairment

*p values were calculated using the Kruskal-Wallis rank test

### Comparison of negative and disinhibition behavior scores between PPA variants

The distribution of scores for PPA variants were significantly different for jocularity (p = 0.02). However, no pairwise group differences were significant after adjustment for multiple comparisons. The distribution of scores for PPA variants were significantly different for restlessness (p = 0.02). After adjusting for multiple comparisons, the scores for lvPPA were lower than those of svPPA. There were no significant differences in the distribution of the remaining disinhibition behaviors or of any of the negative behaviors between the PPA variants.

### Comparison of negative and disinhibition scores within groups

When comparing the difference in negative behavior and disinhibition scores (in percent), the negative percent scores were significantly higher than the disinhibition percent scores in all groups (Table 3).

**Table 3:** Comparison of the difference between negative behavior and disinhibition scores in percent within PPA subtypes and MCI.

Variant	Negative Behavior Percent Score (mean, SD)	Disinhibition Percent Score (mean, SD)	Difference (mean, SD)	P values*
lvPPA (n = 40)	33.23 (21.09)	10.18 (10.73)	23.05 (20.59)	<0.001
nfaPPA (n = 29)	39.72 (24.92)	19.03 (20.61)	20.69 (16.70)	<0.001
svPPA (n = 27)	36.74 (25.46)	16.89 (16.68)	19.85 (16.28)	<0.001
MCI (n = 24)	31.50 (19.04)	16.21 (12.78)	15.29 (13.29)	<0.001

SD, standard deviation; lvPPA, logopenic primary progressive aphasia; nfaPPA, nonfluent agrammatic primary progressive aphasia; svPPA semantic variant primary progressive aphasia; MCI, mild cognitive impairment

*p values were calculated using the Wilcoxon signed rank test

### Comparison of proportion of severity ratings between PPA variants

When comparing severity ratings for the negative behaviors, the proportion of lvPPA having severe personal neglect is 0% compared with 11% in each of the other two PPA variants (p = 0.05). When comparing severity ratings for the disinhibition behaviors, the proportion of lvPPA having severe poor judgment is 0% compared with 14% for the nfaPPA variant (p = 0.04).

### Conclusions

This paper expands knowledge of the non-language behavioral disturbances in PPA subtypes. When comparing the difference in negative behaviors and disinhibition within groups, the percent scores of negative behaviors were higher within all PPA subtypes and MCI. Banks and Weintraub [49] reported that negative behavioral manifestations may be related to mood disturbances, such as depression, which are common in PPA and AD.

The PPA variants appeared to be distinguished by particular behaviors. Restlessness appeared to be more characteristic of svPPA. More severe personal neglect was seen in the svPPA and nfaPPA groups than lvPPA. More severe impairment in judgment distinguished the nfaPPA group from the lvPPA group. These findings are consistent with behavioral manifestations in PPA described previously. For example, in a comparison of svPPA and nfaPPA, those with svPPA were more agitated than those with nfaPPA [42]. Restlessness and agitation may be considered manifestations of a similar behavior along a continuum. In addition, severe impairment of judgment characteristic of nfaPPA in this study may be explained by atrophy of the frontal and insular regions [14, 22, 23] progressing to the dorsolateral prefrontal cortex [24] typical of nfaPPA. The prefrontal cortex is often designated the “cognitive brain” responsible for personality, reasoning, and executive decision-making [53], and the dorsolateral frontal lobe is associated with executive functions [54]. Pathology in this region may result in marked impairments in judgment reported in our nfaPPA group.

The MCI group in our study included primarily amnestic and multidomain subtypes. They had lower total scores for negative behaviors and disinhibition than the PPA variants. Disease duration was not significantly different between the diagnostic groups in our study. However, the more benign behavioral profile of MCI may change with disease progression. MCI differs in etiology and outcome. Amnestic MCI has been shown to progress to AD; non-amnestic MCI has been shown to progress to non-AD dementia [55]. Comparisons of MCI subtypes and PPA variants may reveal important distinctions between these diagnostic groups.

Understanding these behavioral manifestations has clinical and practical implications. Many individuals with PPA and their families/caregivers want to know what to expect in the setting of progressive disease. Extensive research regarding the language profiles of PPA subtypes enables clinicians to counsel patients, families, and caregivers about anticipated communication difficulties. Knowledge of behavioral manifestations in PPA subtypes should enhance counseling efforts, perhaps reducing caregiver burden and facilitating coping. In addition, knowledge of the behavioral manifestations in PPA is important for clinicians in designing treatment that not only addresses language impairments, but also compensates for behavior to facilitate engagement and optimize treatment. While previous studies have highlighted behavioral manifestations in svPPA [35], this study reveals the behavioral disturbances in nfaPPA of equivalent age and duration since onset as well. Limitations of the current study include the sample size and cross sectional study design. Patients were drawn from a convenience sample from one author’s clinical practice using data collected at the initial neurologic consultation. A larger study population may allow investigation of the role of covariates; the patient groups in this study were relatively homogeneous with respect to age and education. Age and education are typically considered in estimating recovery from stroke [56]. Investigation of the role of these factors in PPA and decline is warranted. In addition, studies have not examined gender disparities in decline in PPA, however, gender differences in brain structure in healthy individuals have been reported, supporting the concept of sexual dimorphism in brain structures that may underlie gender differences in behavioral and cognitive functioning and the need to delineate pathophysiological mechanisms underlying sex differences in neuropsychiatric disorders [57]. Finally, although the aim of this study was to distinguish PPA variants, a longitudinal study design would add valuable information about change over time.
